# Precision biomarker discovery in hypertension through explainable AI and proteomics

**DOI:** 10.1038/s41371-026-01134-9

**Published:** 2026-04-22

**Authors:** Karthik Sekaran, Hatem Zayed

**Affiliations:** 1 Bioinformatics Core, Luxembourg Centre for Systems Biomedicine (LCSB), University of Luxembourg, Belvaux, Luxembourg, Luxembourg; 2 Department of Biomedical Sciences, College of Health Sciences, QU Health, Qatar University, Doha, Qatar

**Keywords:** Adrenal gland diseases, Risk factors

## Abstract

Hypertension is a major global health burden and a leading driver of cardiovascular disease, yet reliable blood-based biomarkers for early disease are still limited. We combined plasma proteomics with explainable machine learning to identify circulating proteins associated with stage 1 hypertension in the Qatar Biobank. Proteomic profiles from 778 participants (554 controls and 224 stage 1 hypertension cases) were analyzed; 1305 proteins were tested for differential expression with adjustment for age and sex, and top features were prioritized before training predictive models. Among the evaluated classifiers, CatBoost performed best (AUROC = 0.7985), and SHapley Additive exPlanations were used to interpret the model. We identified 36 proteins significantly associated with hypertension and observed a characteristic pattern featuring lower Renin, sRAGE, ghrelin, and IL-1RAcP, and higher TFPI, QORL1, HSP70, and C5a in hypertensive individuals. Pathway and network analyses implicated processes related to oxidative stress and vascular function. Together, these results demonstrate Renin, TFPI, sRAGE, QORL1, ghrelin, HSP70, IL-1RAcP, and C5a as candidate circulating biomarkers for hypertension and illustrate the value of explainable AI for translating proteomic signals into potentially clinically interpretable candidates, pending validation in independent and diverse cohorts.

## Introduction

Hypertension remains a leading global health burden and a major risk factor for cardiovascular diseases, stroke, and renal complications [1]. Despite its high prevalence, the condition often remains asymptomatic, leading to late diagnosis and increased morbidity [2]. The economic disparities across regions significantly influence hypertension guidelines and treatment accessibility, particularly in low and middle-income countries. Lifestyle factors, including diet, physical activity, stress, smoking, and sleep patterns, are key mediators of hypertension risk [3]. Additionally, demographic variables such as age, sex, socioeconomic status, and genetic predisposition contribute to disease susceptibility [4, 5]. Molecular insights into hypertension etiology have advanced with the rise of multi-omics technologies, providing deeper perspectives into disease mechanisms [6].

Metabolomics studies have linked hypertension to dysregulated metabolic pathways, including alanine, glutamate, and aspartate metabolism [7]. Blood biomarkers such as Renin, Angiotensin A, Leptin, IL-1, and IL-6 are associated with disease onset, while ANP, BNP, nitric oxide, and fibrinogen influence disease progression [8]. Genome-wide association studies (GWAS) have further enhanced understanding by identifying key genetic markers linked to primary hypertension [9]. However, a major challenge remains in translating these findings into clinical diagnostics and personalized medicine.

Recent advances in explainable artificial intelligence (XAI) have enabled the integration of proteomic and clinical data to enhance disease prediction and biomarker discovery [10]. Circulating proteins hold promise as diagnostic markers, offering insights into disease progression and therapeutic responses [11]. Prior studies suggest that the actin cytoskeleton plays a regulatory role in hypertension, highlighting the need for deeper proteomic investigations. Despite extensive research, gaps remain in identifying clinically actionable proteomic biomarkers that can predict hypertension risk with high precision.

We explored proteomic biomarkers for hypertension using the Qatar Biobank cohort, providing valuable insights into the molecular mechanisms underlying blood pressure regulation. [12] conducted a proteomic profiling study using the SOMAscan platform, identifying QORL1 and BMP1 as novel biomarkers and associating them with pathways linked to vascular biology, immune modulation, and atherosclerosis. While we earlier employed statistical association analysis, pathway enrichment, and protein-protein interaction networks, our approach complements and extends these findings by integrating machine learning-driven biomarker identification (HSIC-LASSO), supervised predictive modeling (CatBoost, XGBoost, GBM, LightGBM), and explainability through SHAP analysis. Our approach prioritizes the most predictive proteomic markers and enhances interpretability, allowing for a more robust assessment of hypertension-related signatures. By leveraging AI-driven feature selection and explainability tools, this study aims to refine biomarker identification and advance precision medicine strategies for hypertension.

## Methods

### Study design

The proteomics data provided by the Qatar Precision Health Institute - Qatar Biobank (QPHI-QBB) [13] were analyzed retrospectively. Each proteomic signature was represented as Relative Fluorescence Units (RFU), corresponding to 1305 proteins identified by mapping the aptamer ID to the UniProt ID and Entrez Gene identifiers. The study size included 554 healthy controls and 224 stage 1 hypertension samples. The criteria defining the samples as stage 1 hypertension based on the systolic (130-139 mmHg) and/or diastolic (80-89 mmHg) blood pressure were referred to the guidelines of the American Heart Association (AHA) [14]. Age and sex were two covariates used as adjusting factors in this study because of their relevance to hypertension. Demographic data represent male controls (n = 296; mean age: 37.80), and males with stage 1 hypertension (n = 133; mean age: 46.20). Demographic data represent female controls (n = 258; mean age: 39.22) and females with stage 1 hypertension (n = 91; mean age: 49.29).

### Differential protein expressions

The protein expression levels were estimated based on the RFU values, which are proportional to the abundance of each protein in the sample. Differential protein expression (DPE) analysis was performed using the ‘limma’ R library. Log2 transformation was applied to the protein abundance data to stabilize the variance for understanding relative change in their expression levels. A linear model was fitted to the protein abundance data, adjusted for the covariates age and sex, defined in the design matrix. Statistical estimates were computed using the empirical Bayes method. The top differentially expressed proteins were identified by applying Benjamini-Hochberg (BH) false discovery rate (FDR) adjustment to correct for multiple comparisons (adjusted p-value < 0.05).

### Ranking of proteomic signatures

Hilbert-Schmidt Independence Criterion (HSIC) Least Absolute Shrinkage and Selection Operator (LASSO) algorithm were employed to rank the proteomic signatures identified during differential expression analysis. HSIC measures the dependence between the proteins based on the kernel methods in a higher-dimensional space. LASSO, a widely adopted regression method for feature selection and regularization, controls overfitting by penalization. It adds a penalty term on regression coefficients to shrink to zero, thereby eliminating redundancy in the data. The protein levels often exhibit a non-linear relationship due to their influence by complex biological underpinnings. This method addresses the challenges of non-linearity and multi-collinearity arising in high-dimensional settings.

### Addressing the class imbalance

Out of 778 samples, 554 (71.21%) and 224 (28.79%) represent the control and stage 1 hypertension, respectively. This indicates a significant class imbalance and increases the likelihood of model bias in the results. Oversampling was avoided due to the large difference in sample sizes. Random undersampling was applied to mitigate class imbalance by reducing the number of control samples from 554 to 224. This approach preserved the original measurements of the retained samples while avoiding synthetic data generation.

### Machine learning

The differentially expressed proteomic signatures were used as input features to train four supervised gradient boosting classifiers: eXtreme Gradient Boosting (XGBoost), Gradient Boosting Model (GBM), LightGBM, and CatBoost. Hyperparameters were optimized for each algorithm to maximize predictive performance through the grid search method. Stratified k-fold cross-validation was used for model performance evaluation, ensuring better generalization. Precision, recall, f1-score, and accuracy scores were calculated to evaluate the classifier’s performance.

### Explainability of proteomics data

Interpretability ensures fairness, transparency, and trust in understanding the complex machine learning predictions with their explanations. In this context, the influence of each protein associated with the model prediction for every sample was determined by their importance. The SHapley Additive exPlanations (SHAP) framework was used to interpret the contributions of each protein signature influencing the model predictions. It generates Shapley values for each protein and provides the magnitude of the protein’s contribution and the directionality toward the prediction.

### Ingenuity pathway analysis

Subsequent bioinformatics analysis was performed using the Ingenuity Pathway Analysis (IPA) system. The network and pathway analysis was carried out by mapping the differentially expressed proteins into the IPA database. Adjusted p-value determined the statistically significant proteins for further evaluation. It generated enriched canonical pathways, upstream regulators, disease and functions, regulatory effects, and molecular networks for the significant proteins listed after the cut-off value.

### Protein interactions and functional enrichment analysis

The protein-protein interactions of 36 differentially expressed protein signatures were analyzed using the STRING database. The network was constructed with a confidence threshold of 0.4, including direct and indirect interactions. The functional enrichments were derived from the network for the biological process, cellular component, and molecular function based on the gene ontologies.

### Implementation details

Statistical analysis was carried out with GraphPad Prism (Version 10.4.1). HSIC-LASSO analysis, Machine learning models, and the SHAP framework were implemented in PyCharm (Version 2024.1). Differential protein expression analysis was performed with RStudio (Version 2024.04.2) and R (Version 4.4.1). Ingenuity Pathway Analysis was performed with IPA Software (Qiagen China Co., Ltd., Version: 127006219).

## Results

### Altered protein expression levels

**Fig. 1:**
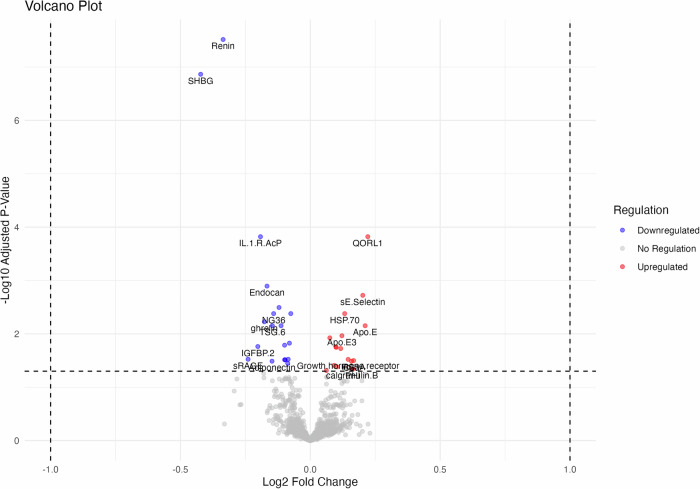
Volcano plot illustrating differentially expressed proteins between stage 1 hypertension and control groups. It is denoted by color gradients (red – upregulated, blue – downregulated, grey – non-significant) based on an adjusted p-value < 0.05. No minimum log2 fold-change threshold was applied to retain biologically relevant signals.

Differential protein expression analysis was conducted using the ‘limma’ R package, with age and sex included as covariates in the design matrix to account for confounding effects. Most proteins exhibited higher statistical significance but a lesser magnitude of change between the conditions (Supplementary Table 1). A total of 1305 proteins were analyzed, with 36 differentially expressed proteins filtered based on the adjusted p-value threshold (FDR < 0.05). The volcano plot (Fig. 1) illustrates the distribution of DEPs, highlighting proteins with significant upregulation and downregulation.

**Fig. 2:**
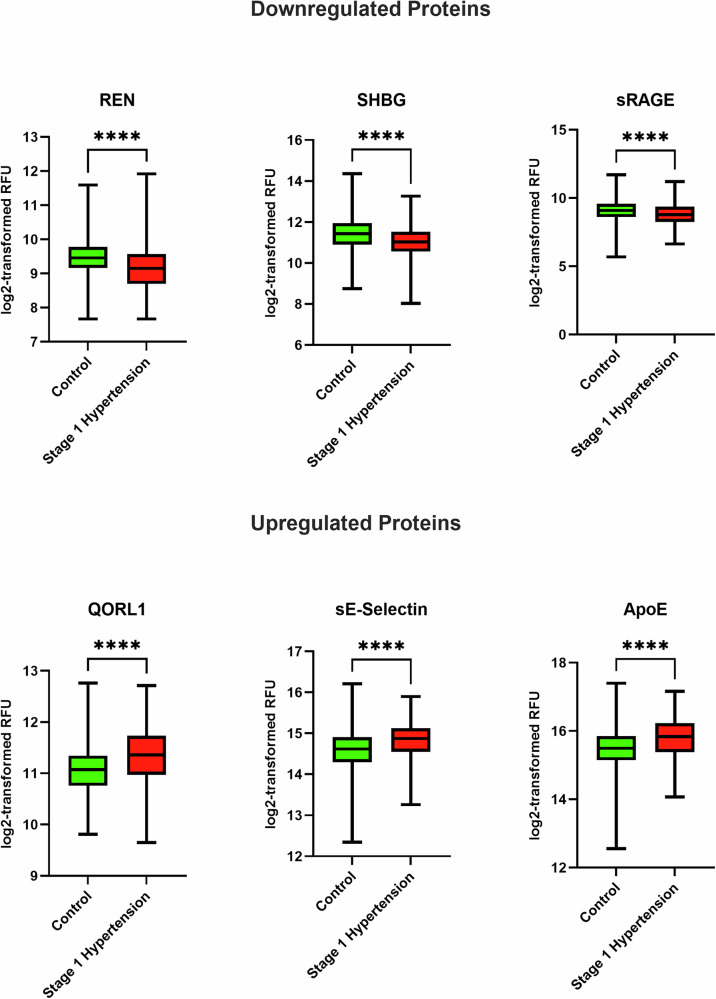
Boxplot of log2-transformed relative fluorescence unit (RFU) values for top 3 up-regulated and top 3 down-regulated proteins. The x-axis denotes comparison groups, while the y-axis represents the log2-transformed RFU values. Statistical significance between the groups was evaluated with the Mann-Whitney U test. p < 0.01 (**), p < 0.001 (***), and p < 0.0001 (****).

Differentially expressed proteins were identified using an adjusted p-value < 0.05 without applying a minimum log2 fold-change threshold, to retain biologically relevant signals. Less stringent criteria were applied (adjusted p-value < 0.05) with no log fold-change considered due to low scores and ensuring the retention of critical biological signals. Applying a |log2 fold change | > 0.25 cut-off yielded only two proteins - Renin (logFC: -0.33) and Sex Hormone-Binding Globulin (SHBG, logFC: -0.42), which were significantly downregulated in hypertensive individuals. In total, 36 DEPs reported after the p-value adjustments were considered statistically significant after multiple testing correction and were used for subsequent bioinformatics and machine learning analyses. The top six DEPs, including Renin, SHBG, sRAGE, QORL1, sE-Selectin, and ApoE, were further visualized in boxplots (Fig. 2), showing distinct expression patterns between hypertensive and control groups. A two-tailed Mann-Whitney U Test was performed to assess statistical significance between hypertensive and control individuals for each DEP.

### Proteome biomarkers of hypertension

The ranking of DEP signatures were calculated using the HSIC-LASSO algorithm. It was directly applied to the DEPs, intended to identify the statistically significant yet exhibiting a non-linearity among the protein markers. This analysis reported the scores for each protein, with Renin (1.0) ranked top, followed by IL-1RAcP (0.51), QORL1 (0.49), TSG-6 (0.47), HSP70 (0.39), sE-Selectin (0.33), SHBG (0.14), ghrelin (0.12), ApoE3 (0.12), and Endocan (0.09).

### Machine learning model performance assessment

**Fig. 3:**
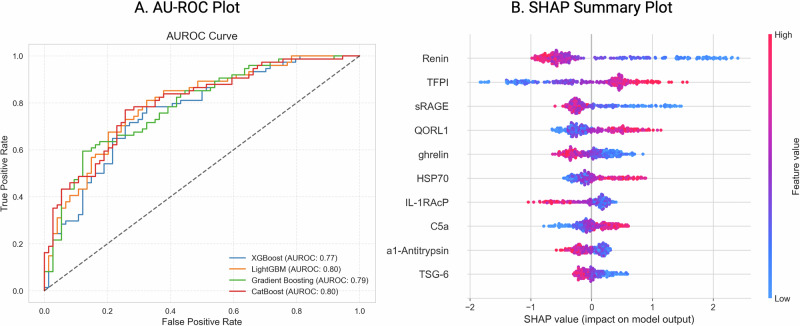
Model performance and feature importance analysis. **A** Receiver Operating Characteristic (ROC) curves comparing the XGBoost, LightGBM, Gradient Boosting Model, and CatBoost for classification performance. **B** SHAP summary plot representing the feature contributions to the CatBoost model output. Higher absolute SHAP values denote a stronger influence of a feature on model predictions.

To assess the predictive capability of the identified differentially expressed proteins, supervised boosting-based machine learning classifiers were trained, including eXtreme Gradient Boosting (XGBoost), LightGBM, Gradient Boosting Model (GBM, implemented using GradientBoostingClassifier from scikit-learn), and CatBoost. The model training was designed systematically to enhance the predictive ability. The dataset was split into a training and testing set (66:34 ratio), ensuring balanced representation using stratified sampling. To mitigate class imbalance, random undersampling was performed on the majority class, which reduced the number of control samples from 554 to 224 to ensure class balancing. It was carried out using the ‘imblearn’ package. Hyperparameter tuning was conducted using a grid search approach, and model validation was performed using five-fold stratified cross-validation to enhance generalizability. The predictive performance of each model was evaluated using AUROC scores, precision, recall, F1-score, and overall accuracy. Among the classifiers, CatBoost achieved the highest AUROC score of 0.7985, indicating superior discriminatory power compared to other models. The detailed performance metrics for all classifiers are summarized in Table 1, while Fig. 3A illustrates the ROC curves for each model. Although LightGBM delivered comparable performance, CatBoost demonstrated a slightly higher AUROC and was therefore selected for biomarker interpretation using SHAP based on proteomic signatures.

**Table 1:** Performance of boosting classifiers.

Model	AU-ROC	Accuracy	Precision	Recall	F1-Score
XGBoost	0.776297	0.709459	0.678161	0.797297	0.732919
LightGBM	0.796497	0.736486	0.716049	0.783784	0.748387
Gradient Boosting	0.787801	0.689189	0.670732	0.743243	0.705128
CatBoost	0.798576	0.736486	0.716049	0.783784	0.748387

### Explanations of the SHAP model on hypertension proteomics data

The CatBoost model predictions were interpreted using the SHAP framework [15] to assess the influence of differentially expressed protein markers on hypertension classification. Global model interpretation was performed using TreeExplainer, which ranked proteins according to their contributions to the model predictions. As illustrated in Fig. 3B, Renin emerged as the most influential biomarker, followed by Tissue Factor Pathway Inhibitor (TFPI), Soluble Receptor for Advanced Glycation End Products (sRAGE), and Quinone Oxidoreductase-Like Protein 1 (QORL1). The SHAP summary plot (Fig. 3B) displays the ranked protein contributions, where the horizontal axis representing the direction and magnitude of impact on the model output. The color gradients indicate feature values, where red representing higher protein levels and blue represents lower protein levels.

Based on SHAP scores, Renin exhibited the higher influence on hypertension classification, reinforcing its well-established role in blood pressure regulation and the renin-angiotensin-aldosterone system (RAAS). TSG-6 and α1-Antitrypsin ranked the lowest among the top 10 most influential proteins, demonstrating minimal contributions, with SHAP values approaching zero. Higher levels of Renin, sRAGE, ghrelin, and IL-1RAcP were generally associated with negative SHAP values, shifting the classifications toward the control class, whereas lower levels of these markers exhibits positive SHAP values, contributing towards hypertension classification. In contrast, higher TFPI, QORL1, and HSP70 were associated with positive SHAP contributions toward hypertension classification, while lower levels shifted classification toward the control group. SHAP values explain each biomarker’s contribution to the model output based on its measured value, rather than implying causality. These findings are consistent with the differential protein expression results (Fig. 2), further supporting the role of these biomarkers in understanding hypertension pathophysiology.

The SHAP summary plot provides a detailed visualization of individual sample contributions, illustrating the distribution of high and low-impact proteins across the dataset. Collectively, these explainability analyses enhance the biological interpretability of machine learning predictions, highlighting the importance of oxidative stress, vascular function, and inflammatory pathways play a critical role in hypertension development.

### Ingenuity pathway analysis

**Fig. 4:**
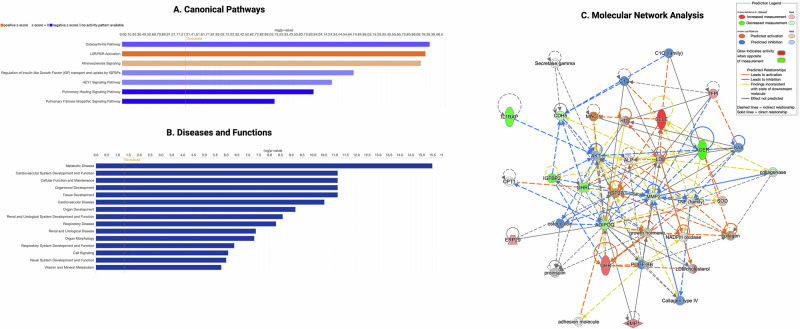
Pathway and molecular network analysis. **A** Canonical pathway enrichment analysis showing significantly altered pathways. The z-score indicates predicted activation (orange) or inhibition (blue). **B** Diseases and biological functions enriched in the dataset, exhibiting key associations with metabolic, cardiovascular and developmental processes. **C** Molecular interaction network depicts relationships among key proteins, with node color indicating expression changes and edges denoting predicted regulatory interactions.

To explore the biological significance of differentially expressed proteins in hypertension, Ingenuity Pathway Analysis was conducted to identify molecular interactions, enriched pathways, and regulatory networks. The canonical pathway analysis (Fig. 4A) identified seven significantly enriched pathways, using a threshold of -log(p-value) > 3 and absolute z-score ≥ 1. Among these, the osteoarthritis pathway exhibited a predicted inhibitory trend (z-score: -1.342; p-value: 5.25e^-07^), suggesting a potential link between inflammatory joint disease and systemic hypertension. The LXR/RXR activation pathway (z-score: 2.00; p-value: 6.41e^-07^) was significantly upregulated, indicating its role in lipid metabolism and vascular homeostasis, while atherosclerosis signaling activation (z-score: 1.00; p-value: 8.01e^-07^) (Fig. 4A).

The Upstream regulator analysis identified 1770 regulatory molecules, including six activators and five inhibitors that influence hypertension-associated pathways. Phosphatase and Tensin Homolog (PTEN) emerged as the top-ranked predicted upstream activator (z-score: 2.183, p-value: 8.72e^-05^), suggesting its role in vascular remodeling and endothelial dysfunction.

Other predicted activators included CFB, RYR1, SB-431542, trans-hydroxytamoxifen, and progesterone, which have established roles in immune modulation, calcium signaling, and hormone regulation. In contrast, Transforming Growth Factor Beta 2 (TGFB2) was the strongest predicted inhibitor (z-score: -2.213, p-value: 6.65e^-06^), suggesting its involvement in fibrosis and vascular remodeling inhibition. Additional inhibitory regulators included CG (complex), prednisolone, deferoxamine, and the estrogen receptor family, underscoring hormonal regulation in hypertension pathogenesis.

The disease and function analysis in IPA (Fig. 4B) revealed that metabolic disease (z-score: 0.204, p-value: 3.16 e^-16^) was the most significantly associated condition, followed by cardiovascular system development and function (z-score: 1.645, p-value: 7.23e^-12^). The regulator effects analysis identified two significant regulatory groups, with consistency scores of 4.914 and 1.342, but no direct regulator-disease function connections were reported.

The molecular network analysis (Fig. 4C) highlighted key protein interactions related to cardiovascular diseases, vascular function, and cellular movement. Five distinct regulatory groups were identified, with the highest-scoring network containing 12 key proteins exhibiting differential expression. Upregulated proteins included BMP1, ERP29, TFPI, GHR, and SELE, while downregulated proteins included CDH5, IL1RAP, MMP2, ADIPOQ, AGER, GHRL, and IGFBO2. These findings reinforce the role of inflammatory and metabolic dysregulation in hypertension pathophysiology, further supporting the biological relevance of identified proteomic signatures.

### Protein interactions and functional enrichment analysis

**Fig. 5:**
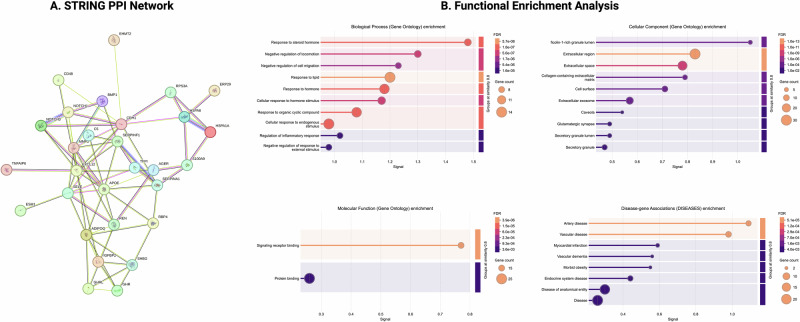
Protein interaction network and functional enrichment analysis. **A** STRING protein–protein interaction (PPI) network of significant proteins, where nodes represent the proteins and edges denote known or predicted interactions. **B** Functional enrichment analysis (Gene Ontology and Disease associations) showing the overrepresented biological processes, cellular components, molecular functions and disease enrichment with significance indicated by FDR and bubble size representing gene counts.

The protein-protein interactions (PPI) network of the 36 DEPs was constructed using STRING database [16] revealing a highly interconnected molecular framework associated with hypertension. The network consisted of 34 nodes and 71 edges, with an average node degree of 4.18, indicating significant functional associations among the identified proteins. The PPI enrichment p-value 6.66e-16 confirmed that the observed interactions were not random, reinforcing the biological relevance of these proteins in hypertension pathophysiology. (Fig. 5A).

Functional enrichment analysis highlighted key biological processes linked to hypertension, including steroid hormone response (GO: 0048545), negative regulation of locomotion (G): 0040013), negative regulation of cell migration (GO: 0030336), response to lipid (GO: 0033993) and response to hormone (GO: 0009725). Only two enriched terms were identified in molecular function: signaling receptor binding (GO: 0005102) and protein binding (GO: 0005515). In cellular components, ficolin-1-rich granule lumen (GO: 1904813), extracellular region (GO: 0005576), collagen-containing extracellular matrix (GO: 0062023), extracellular space (GO: 0005615), and cell surface (GO: 0009986).

Disease-gene associations reported artery disease (DOID: 0050828), vascular disease (DOID: 178), myocardial infarction (DOID: 5844), vascular dementia (DOID: 8725), and morbid obesity (DOID: 11981). All the enrichments scored a false discovery rate < 0.01, other than protein binding (0.0361). The functional enrichment analysis of the STRING network reinforces the influence of the DEPs in vascular functions (Fig. 5B).

## Discussion

### Hypertension-associated proteomic biomarkers: multi-scale insights

This large-scale proteomics study provides novel insights into hypertension-associated protein signatures by integrating machine learning explainability with bioinformatics-driven analyses. Using data from 778 Qatari individuals [12] this study systematically identified 36 differentially expressed proteins with statistical significance, of which Renin, sRAGE, TFPI, QORL1, and HSP70 emerged as the most influential biomarkers. A key strength of this study lies in its multi-layered analytical approach, combining differential protein expression analysis, nonlinear feature selection (HSIC-LASSO), and predictive modeling using boosting-based classifiers (CatBoost, XGBoost, LightGBM, GBM). The explainability assessment using SHAP values further refined biomarker selection, identifying Renin, TFPI, and QORL1 as central drivers of hypertension classification. In parallel, Ingenuity Pathway Analysis and STRING-based protein interaction networks revealed mechanistic insights, linking these biomarkers to vascular function, oxidative stress, and inflammatory pathways. These findings collectively enhance our understanding of the molecular landscape of hypertension, offering potential avenues for biomarker-driven diagnostics and precision medicine interventions.

### Impact of age and sex on protein expression patterns

In this study, age and sex were included as covariates in the design matrix to account for their potential influence on protein expression levels, as both factors are well-documented contributors to hypertension risk and cardiovascular remodeling [17, 18]. Despite the relatively low absolute log2 fold-change threshold ( | 0.2 | ) used in DPE analysis, the study identified 7 proteomic markers with meaningful fold changes, while 36 proteins reached statistical significance (adjusted p-value < 0.05), and 14 remained significant at a stricter threshold (adjusted p-value < 0.01). Notably, Renin, SHBG, sRAGE, and IGFBP-2 were significantly downregulated in hypertensive individuals, whereas QORL1, sE-Selectin, and ApoE exhibited upregulation. These findings align with prior research indicating that reduced Renin levels may reflect compensatory feedback in hypertension pathophysiology [19] while sE-Selectin upregulation is indicative of endothelial activation and inflammation, both of which are hallmarks of vascular dysfunction in hypertensive individuals [20].

### Advanced machine learning models for biomarker discovery

The integration of advanced feature selection and explainable machine learning models has revolutionized the identification of high-confidence proteomic biomarkers for hypertension. In this study, the HSIC-LASSO algorithm provided a nonlinear feature ranking that prioritized Renin, IL-1RAcP, QORL1, TSG-6, and HSP70 as the most influential proteins. This approach outperforms conventional statistical filtering by capturing hidden dependencies within proteomic datasets, aligning with the growing recognition that hypertension is driven by intricate molecular interactions rather than single biomarkers alone [21]. The dominance of Renin in the ranking further reinforces its role in the renin-angiotensin-aldosterone system (RAAS), which remains the most well-established regulator of blood pressure homeostasis. Similarly, the upregulation of QORL1, sE-Selectin, and ApoE in hypertensive individuals supports previous findings linking these proteins to vascular inflammation, endothelial dysfunction, and lipid dysregulation, the key contributors to hypertension pathogenesis [12, 22].

To translate these insights into predictive modeling, boosting-based machine learning classifiers (XGBoost, LightGBM, GBM, CatBoost) were applied to the 36 most significant proteomic markers. While all models exhibited moderate predictive capability, CatBoost demonstrated superior AUROC performance (0.7985) (Fig. 3A). However, this classification score suggests that hypertension is a highly complex condition requiring multi-omics integration for improved predictive accuracy [10]. These findings support emerging research advocating for hybrid models that integrate proteomics with clinical, genomic, and environmental factors to better capture the heterogeneous nature of hypertension risk.

To enhance the interpretability of these predictions, SHapley Additive exPlanations (SHAP) with TreeExplainer were employed, revealing that decreased levels of Renin, sRAGE, and ghrelin, alongside increased TFPI, QORL1, and HSP70, were the most critical contributors to hypertension classification. Although TSG-6 and α1-Antitrypsin exhibited lower SHAP values, their potential functional roles in hypertension-related inflammatory pathways cannot be overlooked. The integration of machine learning explainability tools into biomarker research ensures that these findings are not only statistically significant but also biologically interpretable (Fig. 3B), a critical step toward developing precision medicine applications for hypertension [23].

### Pathway analysis and biological mechanisms of hypertension

The molecular underpinnings of these proteomic markers were scrutinized through ingenuity pathway analysis, to understand enriched pathways and regulatory interactions. Canonical pathway analysis revealed that LXR/RXR activation, was among the most enriched pathways, which plays a key role in inflammation, cholesterol homeostasis, lipid metabolism, and glucose regulation (Fig. 4A) [24]. Atherosclerosis signaling activation was also significantly enriched, supporting the involvement of the identified biomarkers in hypertension pathogenesis (Fig. 4A) [25]. Upstream regulator analysis identified PTEN as a strong activator and TGFB2 as a key inhibitor. While direct evidence linking PTEN to hypertension regulation is limited, previous studies suggest its indirect involvement through vascular remodeling and endothelial function modulation [26]. The association between transforming growth factor-β and hypertension has been extensively studied [27–30]. Progesterone also emerged among the predicted upstream regulators and may contribute to hypertension pathophysiology through hormone-related vascular effects. [31–33]. Conversely, the inhibition of estrogen receptor family proteins plays a crucial role in sex-specific differences in hypertension pathophysiology, as estrogen is known to exhibit protective cardiovascular effects [34, 35].

The STRING protein-protein interaction (PPI) analysis (Fig. 5A & B) further strengthen these findings by identifying key molecular interactions between hypertension-associated proteins. Functional enrichment analysis revealed significant involvement in lipid metabolism, hormone signaling, and steroid hormone response, highlighting the impact of endocrine system dysregulation in hypertension development. More importantly, disease enrichment analysis confirmed that the identified proteomic biomarkers are strongly linked to vascular diseases, further supporting their relevance as potential diagnostic and therapeutic targets.

### SOST: A reliable biomarker or merely another biological signal?

It’s essential to address the impact on the results observed after adjusting the design for the covariates (age and sex). With no adjustments, Sclerostin (SOST), a type of bone cell produced in osteocytes, topped the differentially expressed proteins. It’s upregulated (logFC score: 0.41) with higher statistical significance (adjusted p-value 0.0) [4.89e^-18^]. But, after the adjustment, a high variation was found (logFC score: 0.10, adjusted p-value 0.212), shows no statistical significance. However, some recent findings reported the upregulation of SOST was associated with hypertension and pulmonary complications [36]. The involvement of the RAAS system in the activation of AT1aR upregulates the SOST expressions in osteocytes may be a cause [37]. Disparities in covariate adjustments need careful attention to avoid missing critical biological signals, conversely, not including noise. Further studies on SOST are required to validate the actual association with hypertension.

### Clinical implications and future directions

The multi-level analysis confirmed the pivotal role of Renin, which emerged as the most significant biomarker in this hypertension study. As an initiating enzyme in the renin-angiotensin-aldosterone system (RAAS), Renin plays a central role in blood pressure regulation, vascular resistance, and electrolyte balance. It serves as a critical mediator of vascular, renal, and cardiac physiology, regulating vascular tone, water homeostasis, and sodium retention [38]. Beyond hypertension, Renin is a well-established clinical biomarker for various cardiovascular disorders, including primary aldosteronism [39, 40] and other RAAS-mediated diseases [41–43].

Our findings align with previous clinical evidence demonstrating that lower Renin levels characterize specific subtypes of hypertension, particularly low-renin hypertension. The SHAP model interpretations (Fig. 3B) further corroborated this by showing that decreased Renin levels strongly influence hypertension classification. The significant reduction of Renin levels in hypertensive individuals (Fig. 2) suggests a compensatory feedback mechanism that is frequently observed in low-renin hypertension cases [44–47]. This highlights the necessity of validating proteomic biomarkers using explainable AI models, which enhance model transparency, trust, and clinical applicability.

Beyond Renin, the other identified proteomic markers in this study also exhibit strong clinical relevance to hypertension. Tissue Factor Pathway Inhibitor (TFPI), which was significantly upregulated in hypertensive individuals (Fig. 2), has been previously associated with preeclampsia and endothelial dysfunction [48]. Notably, the upregulation of TFPI in our hypertensive cohort presents a compelling finding. Although some work reported lower TFPI levels in specific subsets of patients with pulmonary hypertension [49], while others have found unchanged levels in other cohorts [50], our observation aligns with findings in other specific hypertensive states, such as preeclampsia, where significantly higher TFPI levels have been observed [48, 51]. Additionally, low levels of Soluble Receptors for Advanced Glycation End Products (sRAGE) were linked to hypertension, cardiovascular diseases, and pulmonary hypertension [52–55]. Likewise, Quinone Oxidoreductase-Like Protein 1 (QORL1) has been implicated in cardiovascular diseases and hypercholesterolemia [56, 57].

Furthermore, Ghrelin, which exhibited a notable reduction in hypertensive individuals (Fig. 2), has been recognized as an essential biomarker for hypertension, atherosclerosis, obesity, and metabolic disorders [58–62]. Similarly, Heat Shock Protein 70 (HSP70), which showed a moderate upregulation (logFC: 0.13, adjusted p-value 0.004), has been implicated in hypertension-related oxidative stress and inflammation [63, 64]. SHAP interpretations (Fig. 3B) reinforced this finding by showing that increased HSP70 levels significantly contributed to hypertension prediction.

Finally, Interleukin-1 Receptor Accessory Protein (IL-1RAcP) has been studied for its potential in reducing hypertension risk through inflammatory pathway modulation. Recent studies suggest that targeting IL-1RAcP could provide cardiovascular protection and reduce atherosclerotic burden. The overall findings strongly align with the SHAP-based feature importance analysis (Fig. 3B), confirming that these proteomic markers play fundamental roles in hypertension pathogenesis. By integrating machine learning explainability with biomarker validation, this study enhances biological interpretation, clinical relevance, and translational potential in hypertension research.

### Cross-population consistency of findings

The proteomic signatures identified in this study show strong concordance with both regional and global hypertension cohorts. A recent QBB-based analysis [12] independently reported Renin, TFPI, and sRAGE as key biomarkers, confirming the internal validity of our AI-based findings. Comparable results have also been observed in diverse populations: Chinese [65], South African [66], German [67], and U.S. [68], where these proteins and related pathways were implicated in vascular inflammation and oxidative stress regulation. This global alignment underscores the biological universality of the identified proteomic biomarkers and supports the potential of our explainable AI framework for cross-cohort validation and precision hypertension modeling.

### Future directions: crosstalk between the proteomic and metabolomic hypertension biomarkers

Trans-Omics is an exciting, emerging field of bioinformatics that integrates multiple omics layers to elucidate biological patterns associated with diseases [69]. It provides a holistic framework for understanding complex molecular data and enables more comprehensive interpretation of potential biological relationships. Recent studies have reported promising results by integrating the phenotypic and molecular data [70, 71]. In the future, integrated multi-omics analysis will be conducted to identify the relationship and the dysregulation mechanism of biological markers at the molecular level. In particular, the interplay between proteomic and metabolomic data may provide more insights into the regulatory mechanisms underlying hypertension.

### Limitations of the study

This is a population-based study that involves participants only among the Qatari population, which introduces selection bias and limit the generalizability of the findings to the populations with different socioeconomic, behavioral, and lifestyle characteristics. Additionally, the lack of external datasets prevented validation in an independent cohort. The dataset exhibited notable class imbalance (554 controls and 224 stage 1 hypertension cases). To mitigate this imbalance, random undersampling was applied to the majority class. Although this approach reduces the total number of available samples, it avoid the introduction of synthetic observations and helps prevent the model from being biased toward the majority class. This strategy was chosen to improve class balance and enhance generalization to unseen data, acknowledging the trade-off between sample size and bias reduction. We focused on boosting-based classifiers, because these methods are well suited for structured tabular data and consistently demonstrated better performance. While SHAP analysis improved the interpretability of the model by identifying influential protein markers, it does not imply causal relationships. While our findings aligns with previously reported hypertension-associated proteomic patterns, external validation in independent and multi-ethnic cohorts remains necessary to confirm the robustness, and cross-population transferability of our model.

## Conclusions

This study presents a comprehensive proteomic analysis of hypertension, identifying Renin, TFPI, sRAGE, QORL1, Ghrelin, HSP70, IL-1RAcP, and C5a as pivotal biomarkers through integrated machine learning and bioinformatics approaches. By leveraging explainable AI models, we established a clear mechanistic link between these proteomic signatures and key hypertension-related pathways, including vascular dysfunction, oxidative stress, and inflammatory signaling. The predictive performance of our models underscores the clinical potential of these biomarkers for early hypertension detection and risk stratification. Moreover, pathway analysis revealed critical regulatory networks that may represent potential therapeutic targets for precision medicine interventions. Despite the molecular complexity of hypertension, our findings provide a foundation for biomarker-driven diagnostics and targeted drug development, advancing the future of AI-integrated, personalized approaches to hypertension management.

## Supplementary information

Supplementary Table

Supplementary Table Legend

## Data Availability

Qatar biobank data can be accessed upon request using the online portal: (https://www.qatarbiobank.org.qa/research/how-apply). This portal is subject to approval by the QBB IRB Committee. This study used the Qatar biobank data under the project (QF-QBB-RES-ACC-00095).
