# Supplementary material for: Precision biomarker discovery in hypertension through explainable AI and proteomics
Source: J Hum Hypertens. 2026 Apr 22;40(6):465–74. doi: 10.1038/s41371-026-01134-9 (PMC13249572; doi:10.1038/s41371-026-01134-9)
Supplement: Supplementary file 2 — Supplementary Table Legend [file 41371_2026_1134_MOESM2_ESM.docx]

**Supporting Information**

**Supplementary Table 1: Differential Protein Expression Analysis**
